# Acute Headache Management and Emergency Department Throughput: A Multicenter Retrospective Analysis

**DOI:** 10.5811/westjem.61415

**Published:** 2026-09-24

**Authors:** Aimen Vanood, Billy J. Evans, Samantha J. Brown, Nicole R. Hodgson, Thom Maciulewicz, Anthony Laine Green, Wayne A. Martini

**Affiliations:** * Mayo Clinic, Department of Neurology, Phoenix, Arizona; † Mayo Clinic, Department of Emergency Medicine, Phoenix, Arizona

## Abstract

**Introduction:**

Headache is a common presentation to the emergency department (ED), requiring both accurate diagnosis and efficient management. Although consensus guidelines exist, real-world treatment practices and their associations with operational outcomes remain variable. We evaluated the association between acute headache management strategies—including medications, diagnostic imaging, and consultations—and ED length of stay (LOS), using both unadjusted and multivariable-adjusted analyses.

**Methods:**

We conducted a multicenter retrospective observational study using data from 79,527 adult ED encounters for headache across 22 EDs in the southwestern and upper Midwestern United States between 2018 and 2024. The primary outcome was ED LOS. Secondary outcomes included LOS differences associated with medication classes, imaging modalities, procedures, and specialty consultations. Length of stay differences by treatment strategy were compared using Mann-Whitney U testing. We performed multivariable linear regression and quantile regression (median) adjusting for age, sex, Emergency Severity Index level, disposition, imaging, procedures, consultations, site, and year. Encounters were classified by headache type (primary, secondary, or unspecified) using discharge diagnosis codes, and stratified analyses were performed.

**Results:**

Primary headache encounters comprised 19.4% of the cohort (*n* = 15,413), secondary headache 14.0% (*n* = 11,147), and unspecified headache 66.6% (*n* = 52,967). The overall median LOS was 183 minutes (interquartile range [IQR], 119–276). In unadjusted analyses, nonsteroidal anti-inflammatory drug (NSAID) (median 180 vs 185 minutes; *P* = .42) and triptan administration (177 vs 183 minutes; *P* = .49) were not associated with longer LOS. In contrast, antiseizure medications (+136 minutes; *P* < .001), intravenous magnesium (+101 minutes; P < .001), opioids (+89 minutes; *P* < .001), and benzodiazepines (+72 minutes; *P* < .001) were associated with the longest LOS. In multivariable-adjusted analysis (R^2^ = .40), antiseizure medications (adjusted coefficient +85.1 min; 95% CI, 76.0–94.3), admission (+71.1 minutes; 95% CI, 68.3–73.8), and magnetic resonance imaging (+125.9 minutes; 95% CI, 119.8–131.9) were most strongly associated with longer LOS. Stratified analyses showed primary headaches had shorter median LOS (153 minutes; IQR, 105–224) than secondary (204 minutes; IQR, 126–317) or unspecified headaches (189 minutes; IQR, 123–282), with consistent medication–LOS associations across subgroups (Kruskal-Wallis H = 1,476.85, *P* < .001; all pairwise comparisons *P* < .001 after Bonferroni correction).

**Conclusion:**

In this multicenter retrospective study, most pharmacologic and diagnostic interventions—aside from NSAIDs and triptans—were associated with longer ED LOS, even after adjusting for patient and clinical covariates. These associations likely reflect both direct treatment effects and confounding by indication. Prospective studies are needed to evaluate whether standardized protocols can improve ED throughput while maintaining care quality.

## INTRODUCTION

Headache is a common complaint in the emergency department (ED), accounting for over three million annual visits in the United States alone.^1^ In 2008, headache comprised the chief complaint of 2.4% of ED visits and was the secondary complaint in 4.4% of ED visits.^1^ For emergency physicians, the challenge lies in rapidly distinguishing life-threatening or time-sensitive secondary causes of headache from primary headache disorders while also delivering effective symptomatic relief and ensuring efficient patient throughput.^2^

Evidence-based management of primary headache disorders, including migraines, tension-type, and cluster headaches, relies on pharmacologic therapies that alleviate pain and reduce recurrence.^2^,^3^ However, red-flag features raise concern for secondary headache, which should prompt further evaluation.^4^

Practices for management of headache in the ED are extrapolated from the migraine literature.^5^ Despite consensus guidelines from organizations such as the American Headache Society (AHS) and the American College of Emergency Physicians (ACEP), variability in prescribing practices remains substantial.^3^,^6^ Medications such as metoclopramide, prochlorperazine, and sumatriptan have demonstrated consistent efficacy and are recommended as first-line agents for acute migraine management.^3^ Furthermore, adjunctive use of dexamethasone has been associated with reduced recurrence rates, while intravenous (IV) acetaminophen has shown promise in enhancing pain control and possibly reducing the use of opioid rescue medications.^7^,^8^ However, adoption of these practices is heterogeneous and heavily influenced by clinician preference, institutional culture, and workflow efficiency. Notably, a survey of subspecialty headache neurologists found that a minority were satisfied with headache management of their patients in the ED.^9^

While prior studies have examined individual aspects of ED headache management, comprehensive multicenter data evaluating the association between diverse treatment strategies and ED throughput remain limited, particularly in the United States.^10^–^12^ Our study aim was to evaluate, at multicenter scale, the association between different headache management strategies and ED length of stay (LOS) using both unadjusted comparisons and multivariable-adjusted analyses.

## METHODS

### Study Design and Setting

We conducted a retrospective observational study using data extracted from the electronic health record (EHR) (Epic Systems) across multiple EDs. These included the three tertiary care sites in Phoenix, Arizona, Jacksonville, Florida, and Rochester, Minnesota, as well as 19 community hospitals spanning Minnesota and Wisconsin. This study was deemed exempt by the institutional review board. This study adheres to the Strengthening the Reporting of Observational Studies in Epidemiology (STROBE) reporting guideline.

**Population Health Research Capsule**

What do we already know about this issue?

*Headache is a common emergency department (ED) chief complaint, but real-world associations between treatment choice and length of stay vary widely*.

What was the research question?

*Do specific acute headache treatments increase ED length of stay (LOS), after adjusting for patient and clinical factors?*

What was the major finding of the study?

*Antiseizure medications added 85 minutes to ED LOS (95% CI, 76*–*94 min; P < .001) vs nonsteroidal anti-inflammatory drugs and triptans (no association)*.

How does this improve population health?

*Identifying which acute headache treatments increase LOS can guide protocols that improve ED throughput without compromising care*.

### Study Population

Data extraction was performed from the Clarity reporting database via structured query language through the institution’s unified data pool. Initially, 2,906,593 ED encounters across all sites were identified between January 1, 2018–December 31, 2024. After excluding pediatric patients (< age 18), 2,481,384 adult encounters remained. Headache-related visits were identified by querying both structured diagnosis fields and reason-for-visit entries. Encounters were included if the reason for visit matched “Migraine” or “Headache,” or if they carried diagnoses matching headache-related terms including “headache,” “migraine,” “subarachnoid,” “intracerebral hemorrhage,” “intracranial hemorrhage,” “subdural,” “epidural,” “hematoma,” “meningitis,” “encephalitis,” “venous thrombosis,” “arteritis,” “hypertensive encephalopathy,” “intracranial hypertension,” “pseudotumor,” “intracranial hypotension,” or “CSF leak.” This methodology ensured the comprehensive inclusion of primary headache disorders as well as secondary neurological conditions presenting with headache symptoms. We excluded visits reporting trauma on computed tomography (CT) imaging indication or with a diagnosis linked to trauma and EHR test patients. The initial cohort comprised 79,770 patient encounters.

### Headache Classification

Encounters were classified into three groups based on discharge diagnosis codes (DX_NAME): (1) primary headache (migraine, tension-type, cluster, benign headache, and related diagnoses; *n* = 15,413); (2) secondary headache (intracranial hemorrhage, subdural hematoma, subarachnoid hemorrhage, stroke, meningitis, encephalitis, sinusitis, concussion, head injury, COVID-19, and other secondary etiologies; *n* = 11,147); and (3) unspecified headache (headache unspecified, headache not otherwise specified, and diagnoses without clear primary or secondary classification; *n* = 52,967). This stratification was performed to address the potential confounding introduced by diagnostic heterogeneity.

### Outcome Measures

The primary outcome was ED LOS, defined as the time from ED arrival to ED departure. Encounters with LOS ≤ 0 minutes (*n* = 44) or > 1,440 minutes (24 hours; *n* = 199) were excluded, yielding an analytic sample of 79,527 encounters. Sensitivity analyses using a 720-minute (12-hour) threshold were also performed.

### Data Collection

Clinical and demographic information—including Emergency Severity Index (ESI), age, sex, race, ethnicity, LOS, and disposition—was extracted from encounter-level tables. Medication administration was restricted to active orders administered during the ED stay, operationalized by requiring that the medication order start time fell between the arrival and departure times. Imaging, consultations, and procedures, including CT, magnetic resonance imaging (MRI), neurology consultation, nerve blocks, and lumbar punctures, were filtered as completed between arrival and disposition time to ensure they reflected the emergency workup.

### Statistical Analysis

Descriptive statistics were reported as medians with interquartile ranges (IQR) for continuous variables and frequencies with percentages for categorical variables. We compared LOS distributions for each medication using the Mann-Whitney *U* test. A two-sided *P* value < .05 was considered statistically significant.

Multivariable linear regression (ordinary least squares) was performed with LOS as the dependent variable. Covariates included all 13 medication classes (binary), patient age, sex, ESI level (categorical; reference, ESI 3), disposition (discharged vs admitted/observed/transferred), CT performed, MRI performed, neurology consultation, lumbar puncture, site (categorical, 22 sites), and year. Quantile regression at the median (q = 0.50) was also performed to provide estimates robust to the skewed LOS distribution.

We performed stratified analyses by headache type (primary, secondary, unspecified), including both Mann-Whitney *U* tests and multivariable regression within the primary headache subgroup. Sensitivity analyses included the following: (1) restricting to discharged patients only; and (2) using a 720-minute LOS exclusion threshold. Analyses were performed using Python 3 with pandas, SciPy, and statsmodels.

### Missing Data

Emergency Severity Index values were missing for 2,839 encounters (3.6%) in the analytic sample; these were excluded from regression analyses (regression *n* = 76,688). All other variables had complete data.

## RESULTS

### Study Population

After excluding encounters with LOS ≤ 0 or > 1,440 minutes (n = 243), the analytic sample comprised 79,527 encounters. Demographic information is summarized in Table 1. Patients of female sex comprised 66.4% of the cohort. The mean age was 48.0 years (SD 19.8), and median age was 45.0 years. By headache classification, 15,413 (19.4%) had primary headache diagnoses, 11,147 (14.0%) had secondary headache diagnoses, and 52,967 (66.6%) had unspecified headache diagnoses.

### Medication Use Trends

Medication administration patterns over the seven-year period are shown in Table 2. Intravenous fluids (43–48%), nonsteroidal anti-inflammatory drugs (NSAID) (37–42%), and dopamine antagonists (27–31%) consistently ranked as the most frequently administered treatments. Antiseizure medications were the least frequently administered (0.26–1.50%), along with IV magnesium (0.73–2.60%) and triptans (1.23–2.06%).

Annual use trends (Figure 1) demonstrated relative stability for most medications. Notable trends included the following: Acetaminophen use increased from 14.8% to 29.1%; IV magnesium grew from 0.73% to 2.60%; and antiseizure medication administration increased nearly six-fold from 0.26% to 1.50%. Opioid administration remained stable (11.2–11.7%). Diphenhydramine use declined from 31.0% to 26.7%.

Medication use differed substantially by headache type: Primary headache encounters had higher rates of dopamine antagonists (50.1% vs 9.8% secondary), NSAIDs (64.2% vs 14.1%), diphenhydramine (52.5% vs 7.6%), and triptans (4.1% vs 0.2%), while secondary headache encounters had higher rates of opioids (20.0% vs 6.4%), acetaminophen (26.5% vs 13.4%), antihypertensives (15.6% vs 1.0%), and antiseizure medications (3.2% vs 0.2%).[Fig f2-wjem-27-1436]

### Imaging and Procedural Trends

A summary of imaging studies, lumbar punctures, nerve blocks, and neurology consultations is shown in Table 3. Computed tomography use rose from 37.9% in 2018 to 51.2% in 2024. Magnetic resonance imaging use increased from 0.95% to 2.95%. Lumbar puncture rates remained stable (~1.2%). Neurology consultations increased three-fold, from 2.3% in 2018 to 6.9% in 2024.[Fig f3-wjem-27-1436]

### Length of Stay: Unadjusted Medication Associations

The overall median LOS was 183 minutes (interquartile range [IQR], 119–276). For encounters without any medication, imaging, procedure, or consultation, the baseline median LOS was 106 minutes (IQR, 62–172). Table 4 presents the corrected Mann-Whitney *U* test results comparing median LOS by medication. Administration of NSAIDs (median 180 vs 185 minutes; difference, −5 min; *P* = .42) and triptans (177 vs 183 min; difference, −6 minutes; *P* = .49) were not associated with longer LOS. Diphenhydramine showed a statistically significant but clinically negligible difference (182 vs 183 minutes; difference, −1 minute; *P* < .001), likely driven by the large sample size.

Antiseizure medications were associated with the greatest LOS difference (+136 minutes; median 318 vs 182 minutes; *P* < .001), followed by IV magnesium (+101 minutes; *P* < .001), opioids (+89 minutes; *P* < .001), benzodiazepines (+72 minutes; *P* < .001), antihypertensives (+69 minutes; *P* < .001), and acetaminophen (+68 minutes; *P* < .001). Detailed results including IQR for each comparison are presented in Table 4.

### Length of Stay: Multivariable-Adjusted Analysis

The multivariable ordinary least squares regression model (*N* = 76,688; *R*^2^ = .40) is presented in Table 5. After adjusting for age, sex, ESI, disposition, imaging, procedures, consultations, site, and year, the medications most strongly associated with longer LOS were antiseizure medications (+85.1 minutes; 95% CI, 76.0–94.3; *P* < .001), acetaminophen (+40.9 minutes; 95% CI, 38.8–42.9; *P* < .001), benzodiazepines (+38.6 minutes; 95% CI, 35.1–42.1; *P* < .001), and opioids (+37.5 minutes; 95% CI, 34.7–40.2; *P* < .001).

Triptans showed a notable adjusted association (+34.6 minutes; 95% CI, 27.8–41.4; *P* < .001), which contrasts with the unadjusted null finding and likely reflects residual confounding: triptan recipients tend to be less acutely ill (fewer CT, lower admission rates), and adjusting for these factors unmasks the time associated with triptan administration itself. Nonsteroidal anti-inflammatory drugs showed a small but significant adjusted association (+4.7 minutes; 95% CI, 2.7–6.7; *P* < .001). Diphenhydramine was not independently associated with LOS after adjustment (+1.3 minutes; 95% CI, −0.9 to 3.5; *P* = .25).

Among nonmedication covariates, hospital admission was associated with +71.1 minutes (95% CI, 68.3–73.8), lumbar puncture with +153.5 minutes (95% CI, 146.2–160.8), MRI with +125.9 minutes (95% CI, 119.8–131.9), CT with +44.2 minutes (95% CI, 42.4–46.0), and neurology consultation with +41.2 minutes (95% CI, 37.3–45.1).

Quantile regression at the median (pseudo *R*^2^ = .27) confirmed similar patterns: antiseizure medications (+40.2 minutes), IV fluids (+31.3 minutes), opioids (+29.7 minutes), IV magnesium (+28.3 minutes), and acetaminophen (+27.4 minutes) had the largest adjusted median effects. Diphenhydramine showed a small significant median effect (+2.8 minutes; *P* < .001) but was of negligible clinical magnitude.

### Length of Stay: Imaging and Procedures

Median LOS for imaging and procedures are summarized in Table 6. Patients without imaging, procedures, or consultation had a median LOS of 145 minutes. Computed tomography imaging was associated with a median of 228 minutes, MRI with 348–399 minutes, lumbar puncture with 444 minutes, and neurology consultation with 293 minutes. The association between imaging use and LOS is expected, as imaging inherently adds to the ED workup duration.[Fig f4-wjem-27-1436][Fig f5-wjem-27-1436]

### Stratified Analysis by Headache Type

Primary headache encounters (*n* = 15,413) had a median LOS of 153 minutes (IQR, 105–224), compared with 204 minutes (IQR, 126–317) for secondary headaches (*n* = 11,147) and 189 minutes (IQR, 123–282) for unspecified headaches (*n* = 52,967). Within the primary headache subgroup, the direction and rank order of medication–LOS associations were generally consistent with the full cohort. Intravenous magnesium (+96 min), opioids (+68 minutes), benzodiazepines (+65 minutes), and acetaminophen (+62 minutes) remained the medications most strongly associated with longer LOS. Nonsteroidal anti-inflammatory drugs (+5 minutes; *P* < .001) and triptans (+7 minutes; *P* = .09) were not associated with clinically meaningful differences.

In the multivariable regression restricted to primary headaches (n = 14,634; *R*^2^ = .47), opioids (+33.6 minutes; 95% CI, 28.0–39.2), triptans (+30.9 minutes; 95% CI, 24.3–37.6), IV magnesium (+30.2 minutes; 95% CI, 22.3–38.0), and acetaminophen (+30.3 minutes; 95% CI, 26.3–34.3) were the strongest independent medication predictors. Nonsteroidal anti-inflammatory drugs were not significant (−0.5 minutes; *P* = .74). Antiseizure medications were not significant in this subgroup (+10.4 minutes; *P* = .50), likely due to very small numbers (*n* = 29). Full stratified results are provided in the Supplemental Tables.

### Sensitivity Analyses

Results were robust across sensitivity analyses. When restricted to discharged patients only (*n* = 64,559; median LOS 170 minutes), the rank order of medication–LOS associations was preserved, with antiseizure medications (+124 minutes), IV magnesium (+91 minutes), and opioids (+68 minutes) remaining the top three. When using a 720-minute (12-hour) LOS exclusion threshold (*n* = 78,612), results were virtually identical to the primary analysis.

## DISCUSSION

Our analytic sample of 79,527 adult ED encounters for headache across 22 EDs over a seven-year period represents, to our knowledge, the largest reported cohort evaluating headache management and ED LOS. The overall median LOS of 183 minutes falls within the range reported in prior published cohorts (186–381 minutes).^10^–^13^ Our findings are also broadly consistent with a nonpeer-reviewed ACEP Research Forum abstract by Wang, which reported that route of administration and drug class were each correlated with LOS in primary-headache ED visits in a single-institution cohort.^14^ The convergence of medication–LOS associations across that independent single-institution dataset and our multicenter cohort strengthens the inference that these patterns are not artifacts of any single institution’s case-mix or operational model. We found that most pharmacologic interventions, aside from NSAIDs and triptans, were associated with longer LOS in both unadjusted and multivariable-adjusted analyses. Importantly, our cohort included a heterogeneous mix of primary headaches (19.4%), secondary headaches (14.0%), and unspecified headache diagnoses (66.6%), which influences the interpretation of these findings.

### Medication–Length of Stay Associations

The most commonly administered therapies—IV fluids, NSAIDs, and dopamine antagonists—largely align with current consensus guidelines.^3^,^13^,^15^ Nonsteroidal anti-inflammatory drug administration was not associated with longer LOS in unadjusted analysis and showed only a small adjusted association (+4.7 minutes), consistent with their role as first-line agents for acute headache. Dopamine antagonists showed a modest unadjusted association (+18 minutes) that attenuated in the adjusted model (+13.2 minutes), supporting their use as efficient first-line therapy. Randomized trials have demonstrated that prochlorperazine with diphenhydramine results in sustained headache relief with less likelihood of headache recurrence within 48 hours compared to hydromorphone^16^ and faster reduction in pain intensity compared to sumatriptan.^17^

Diphenhydramine, often co-administered with dopamine antagonists, showed no clinically meaningful unadjusted difference (−1 minute) and was not significant after adjustment (+1.3 minutes; *P* = .25). This is consistent with evidence suggesting that diphenhydramine does not significantly impact headache outcomes.^18^,^19^

Antiseizure medications, opioids, benzodiazepines, and IV magnesium were associated with the longest LOS in both unadjusted and adjusted analyses. However, these associations must be interpreted cautiously. Patients receiving these medications likely represent more severe or refractory presentations requiring treatment escalation, and thus these medications may serve as markers of clinical complexity rather than independent drivers of prolonged stays. This interpretation is supported by the observation that admission status—a proxy for clinical severity—was independently associated with +71.1 minutes of LOS in the adjusted model.

Opioid use remained stable over the study period (11.2–11.7%), which compares favorably to national datasets from 2010 reporting opioid use in 49–59% of headache-related ED visits.^20^,^21^ Both the AHS and ACEP guidelines recommend against routine opioid use for acute headache.^3^,^6^ Similarly, benzodiazepine use remained stable at approximately 6%, and current guidelines recommend against their use for acute headache treatment.^3^,^6^

### Imaging and Consultations

Diagnostic testing and consultations were associated with progressively longer LOS, as expected given the inherent time required for these evaluations. Computed tomography use rose from 37.9% to 51.2% over the study period, a trend consistent with broader patterns of increasing neuroimaging use.^6^,^13^,^22^,^23^ Notably, higher imaging and consultation rates may be clinically appropriate, particularly among the 14% of encounters with secondary headache diagnoses (hemorrhage, stroke, meningitis), where advanced workup is indicated.

An important contextual factor was the West Nile virus outbreak in Maricopa County, Arizona, between May and December 2021, which likely increased lumbar puncture, neuroimaging, and neurology consultation rates during that period.^24^

### Stratified Findings

Primary headache encounters had shorter median LOS (153 minutes) and higher rates of guideline-concordant medications (dopamine antagonists 50.1%, NSAIDs 64.2%), while secondary headache encounters had longer LOS (204 minutes) and higher rates of opioids (20.0%), antihypertensives (15.6%), and antiseizure medications (3.2%). These differences underscore the importance of stratification when interpreting LOS associations in a diagnostically heterogeneous cohort.

### Adjusted vs. Unadjusted Results

The multivariable model (*R*^2^ = .40) explained a substantial proportion of LOS variability and provides a more accurate picture of independent medication–LOS associations. Several notable differences emerged between unadjusted and adjusted results: triptans showed no unadjusted difference but a significant adjusted association (+34.6 minutes), likely reflecting that triptan recipients are selected lower acuity patients whose shorter raw LOS masks the actual time associated with administration. Conversely, the large unadjusted associations for opioids and antiseizure medications attenuated in the adjusted model (from +89 to +37.5 minutes for opioids; from +136 to +85.1 minutes for antiseizure medications), confirming substantial confounding by indication.

### Future Directions

Prospective studies and quality improvement initiatives evaluating standardized headache management protocols are needed to determine whether observed LOS differences can be reduced while maintaining or improving patient-centered outcomes including pain relief, recurrence rates, and patient satisfaction—not only operational throughput.

## LIMITATIONS

This study has several important limitations. First, the retrospective observational design precludes causal inference. Observed associations between medications and LOS may reflect confounding by indication: Patients with more severe or refractory headaches receive more interventions and independently have longer stays. While multivariable adjustment mitigates this bias, unmeasured confounders (eg, pain severity, comorbidities, prior ED visits) may persist. Second, our cohort includes a heterogeneous mix of primary headache, secondary headache, and diagnostically unspecified encounters. Although we performed stratified analyses, the large proportion of unspecified diagnoses (66.6%) limits the precision of headache-type classification. Medication and imaging patterns that appear discordant with primary headache guidelines may be clinically appropriate for secondary or undifferentiated headache presentations.

Third, potential timing bias exists: Medications administered later in the ED course (eg, as rescue therapy or after failed initial treatment) will inherently be associated with longer LOS, even if they do not independently prolong the stay. Medication timing data (time to first medication) were not available in our dataset. Fourth, we did not adjust for ED crowding, boarding, or staffing levels, which are known to affect LOS independently of clinical factors. Fifth, confounding variables including patient acuity beyond ESI, comorbid conditions, and concurrent treatments were not fully adjusted for. Medications may serve as proxies for more complex clinical presentations. Additionally, we did not examine whether patients were established with outpatient neurology consultants with existing care plans.

Finally, due to the nonrandomized nature of medication administration, observed associations may not accurately reflect causal relationships. However, the multicenter design, large sample size, seven-year study period, and the addition of multivariable-adjusted analyses enhance the validity and applicability of our findings.

## CONCLUSION

In this multicenter retrospective study of 79,527 ED headache encounters, most pharmacologic interventions—aside from NSAIDs and triptans—were associated with longer LOS in both unadjusted and multivariable-adjusted analyses. These associations likely reflect a combination of direct treatment effects and confounding by indication. Prospective studies evaluating standardized headache management protocols and their effects on both operational and patient-centered outcomes are needed.

## Supplementary Information



## Figures and Tables

**Figure 1:**
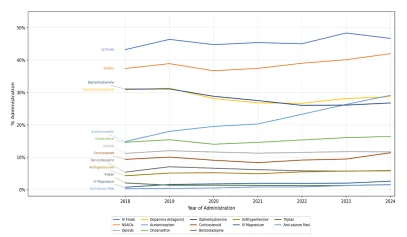
Annual use trends stratified by medication class over the seven-year study period.

**Figures 2a–b f2-wjem-27-1436:**
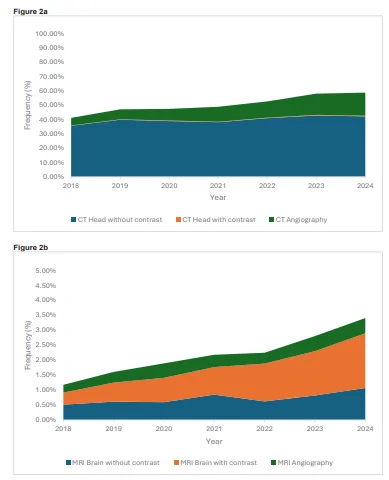
Trends in CT use (Figure 2a) and MRI use (Figure 2b) over the seven-year study period. Note the vertical axis for Figure 2b has a maximum of 5%, given the low frequency of use in our emergency department, in a study of emergency department evaluation and management of headache from 2018–2024.

**Figure 3 f3-wjem-27-1436:**
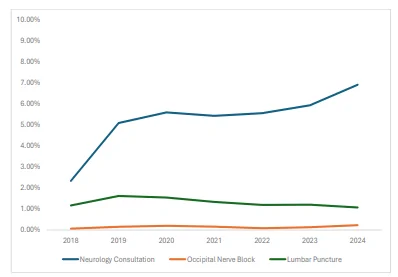
Trends in lumbar puncture, nerve block, and inpatient neurology consultation over the seven-year study period, in a study of emergency department evaluation and management of headache from 2018–2024.

**Figure 4 f4-wjem-27-1436:**
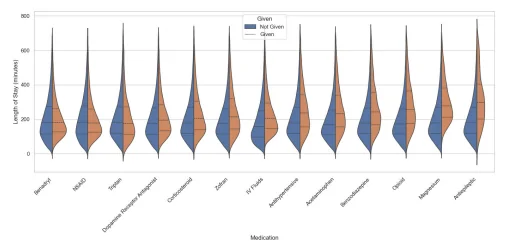
Violin plot of the distribution of length of stay, stratified by whether the medication was given (orange) or not given (blue), in a study of emergency department management of headache from 2018–2024.

**Figure 5 f5-wjem-27-1436:**
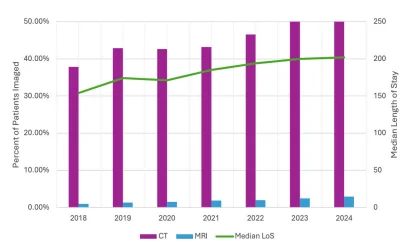
Bar–line chart depicting the correlation between computed tomography and magnetic resonance imaging and median length of stay, in a study of emergency department evaluation and management of headache from 2018–2024. *CT*, computed tomography; *LOS*, length of stay; *MRI*, magnetic resonance imaging.

**Table 1:** Patient demographics in a study evaluating the association between acute headache management strategies and length of stay in the emergency department.

Characteristic	Total patients (%) (*N* = 79,770)
Age, Years	
Mean (SD)	48.04 (19.81)
Median (IQR)	45.00 (32)
Biologic Sex, *n* (%)	
Male	26,753 (33.54%)
Female	53,003 (66.44%)
Nonbinary	7 (0.01%)
Unknown	7 (0.01%)
Race, *n* (%)	
American Indian or Alaska Native	670 (0.84%)
Asian	1,824 (2.29%)
Black or African American	5,835 (7.31%)
Native Hawaiian or Pacific Islander	217 (0.27%)
White	68,197 (85.49%)
Unknown or not reported	3,027 (3.79%)
Ethnicity, *n* (%)	
Hispanic or Latino	6,265 (7.85%)
Not Hispanic or Latino	71,877 (90.11%)
Unknown or not reported	1,628 (2.04%)

*IQR*, interquartile range; *SD*, standard deviation.

**Table 2:** Trends in medication administration (2018–2024) in a study evaluating the association between acute headache management strategies and length of stay in the emergency department.

Medication Category	2018 (*n* = 9,187)	2019 (*n* = 11,755)	2020 (*n* = 9,925)	2021 (*n* = 11,918)	2022 (*n* = 11,917)	2023 (*n* = 12,076)	2024 (*n* = 12,992)	Total (%)
IV Fluids	3,969 (43.20%)	5,450 (46.36%)	4,441 (44.75%)	5,408 (45.38%)	5,364 (45.01%)	5,837 (48.34%)	6,062 (46.66%)	36,531 (45.59%)
NSAIDs	3,434 (37.38%)	4,569 (38.87%)	3,640 (36.68%)	4,460 (37.42%)	4,647 (38.99%)	4,841 (40.09%)	5,449 (41.94%)	31,040 (37.48%)
Dopamine Antagonists	2,834 (30.85%)	3,679 (31.30%)	2,789 (28.10%)	3,190 (26.77%)	3,174 (26.63%)	3,397 (28.13%)	3,720 (28.63%)	22,783 (28.42%)
Diphenhydramine	2,850 (31.02%)	3,653 (31.08%)	2,858 (28.80%)	3,274 (27.47%)	3,099 (26.00%)	3,150 (26.08%)	3,474 (26.74%)	22,358 (27.90%)
Acetaminophen	1,363 (14.84%)	2,112 (17.97%)	1,936 (19.51%)	2,414 (20.26%)	2,771 (23.25%)	3,174 (26.28%)	3,779 (29.09%)	17,549 (21.89%)
Ondansetron	1,341 (14.60%)	1,809 (15.39%)	1,390 (14.01%)	1,738 (14.58%)	1,826 (15.32%)	1,937 (16.04%)	2,132 (16.41%)	12,173 (15.19%)
Opioids	1,027 (11.18%)	1,412 (12.01%)	1,152 (11.61%)	1,340 (11.24%)	1,367 (11.47%)	1,416 (11.73%)	1,513 (11.65%)	9,227 (11.83%)
Corticosteroids	857 (9.33%)	1,179 (10.03%)	900 (9.07%)	992 (8.32%)	1,087 (9.12%)	1,139 (9.43%)	1,476 (11.36%)	7,630 (9.52%)
Benzodiazepines	496 (5.40%)	829 (7.05%)	655 (6.60%)	734 (6.16%)	691 (5.80%)	687 (5.69%)	763 (5.87%)	4,855 (6.06%)
Antihypertensives	392 (4.27%)	600 (5.10%)	517 (5.21%)	584 (4.90%)	652 (5.47%)	692 (5.73%)	752 (5.79%)	4,189 (5.23%)
Magnesium	67 (0.73%)	185 (1.57%)	175 (1.76%)	231 (1.94%)	228 (1.91%)	239 (1.98%)	338 (2.60%)	1,463 (1.83%)
Triptans	189 (2.06%)	158 (1.34%)	136 (1.37%)	154 (1.29%)	147 (1.23%)	154 (1.28%)	202 (1.55%)	1,140 (1.42%)
Antiseizure medications	24 (0.26%)	44 (0.37%)	49 (0.49%)	99 (0.83%)	98 (0.82%)	152 (1.26%)	195 (1.50%)	661 (0.82%)

*IV*, intravenous; *NSAID*, nonsteroidal anti-inflammatory drug.

**Table 3:** Imaging studies and procedures performed (2018–2024) in a study evaluating the association between acute headache management strategies and length of stay in the emergency department.

Year	CT (Any)	MRI	Lumbar puncture	Nerve blocks	Neurology consultation
2018 (*n* = 9,187)	3,477 (37.8%)	87 (0.9%)	107 (1.2%)	6 (0.1%)	214 (2.3%)
2019 (*n* = 11,755)	5,044 (42.9%)	157 (1.3%)	190 (1.6%)	18 (0.2%)	597 (5.1%)
2020 (*n* = 9,925)	4,234 (42.7%)	150 (1.5%)	153 (1.5%)	20 (0.2%)	554 (5.6%)
2021 (*n* = 11,918)	5,145 (43.2%)	224 (1.9%)	159 (1.3%)	19 (0.2%)	646 (5.4%)
2022 (*n* = 11,917)	5,546 (46.5%)	235 (2.0%)	142 (1.2%)	10 (0.1%)	661 (5.5%)
2023 (*n* = 12,076)	6,143 (50.9%)	292 (2.4%)	145 (1.2%)	16 (0.1%)	715 (5.9%)
2024 (*n* = 12,992)	6,646 (51.2%)	383 (2.9%)	139 (1.1%)	30 (0.2%)	896 (6.9%)
Total (%)	36,235 (45.42%)	1,528 (1.92%)	1,035 (1.30%)	119 (0.15%)	4,283 (5.37%)

*CT*, computed tomography; *MRI*, magnetic resonance imaging.

**Table 4:** Unadjusted associations between medication administration and emergency department length of stay.

Medication	*n* given	Median LOS given (IQR)	*n* not given	Median LOS not given (IQR)	Difference (min)	*P* value
Antiseizure medications	632	318 (213–499)	78,895	182 (119–274)	+136	< .001
IV magnesium	1,458	282 (213–389)	78,069	181 (118–273)	+101	< .001
Opioids	9,167	263 (178–383)	70,360	174 (114–261)	+89	< .001
Benzodiazepines	4,807	251 (172–376)	74,720	179 (117–269)	+72	< .001
Antihypertensives	4,105	249 (161–372)	75,422	180 (117–270)	+69	< .001
Acetaminophen	17,421	238 (159–355)	62,106	170 (112–255)	+68	< .001
IV fluids	36,444	207 (146–299)	43,083	159 (96–254)	+48	< .001
Ondansetron	12,110	219 (145–331)	67,417	177 (115–266)	+42	< .001
Corticosteroids	7,598	208 (141–311)	71,929	180 (117–272)	+28	< .001
Dopamine antagonists	22,731	196 (133–291)	56,796	178 (113–270)	+18	< .001
Diphenhydramine	22,315	182 (126–266)	57,212	183 (116–280)	−1	< .001
NSAIDs	31,001	180 (124–263)	48,526	185 (115–284)	−5	.42
Triptans	1,136	177 (113–280)	78,391	183 (119–276)	−6	.49

Results of the Mann-Whitney U test comparing emergency department (ED) length of stay (LOS) by medication administration status. Median LOS with interquartile range (IQR) is shown for encounters where the medication was given versus not given. Difference is the median LOS when given minus the median LOS when not given. A positive difference indicates longer LOS when the medication was administered. Analytic sample: *N* = 79,527 encounters with LOS > 0 and at most 1,440 minutes, in a study of ED management of headache from 2018–2024.

*IQR*, interquartile range; *IV*, intravenous; *LOS*, length of stay; *min*, minutes; *NSAID*, nonsteroidal anti-inflammatory drug.

**Table 5:** Multivariable-adjusted associations between emergency department interventions and length of stay.

Panel A: Medication coefficients						

Variable	OLS Coefficient (min)	95% CI	*P* value	QR Coefficient (min)	95% CI	*P* value
Antiseizure medications	+85.1	76.0, 94.3	< .001	+40.2	33.1, 47.3	< .001
Acetaminophen	+40.9	38.8, 42.9	< .001	+27.4	25.8, 28.9	< .001
Benzodiazepines	+38.6	35.1, 42.1	< .001	+26.6	23.9, 29.3	< .001
Opioids	+37.5	34.7, 40.2	< .001	+29.7	27.6, 31.9	< .001
Triptans	+34.6	27.8, 41.4	< .001	+22.0	16.8, 27.3	< .001
IV Magnesium	+32.4	26.1, 38.6	< .001	+28.3	23.5, 33.2	< .001
IV Fluids	+30.3	28.3, 32.3	< .001	+31.3	29.7, 32.8	< .001
Antihypertensives	+28.2	24.3, 32.2	< .001	+11.9	8.9, 15.0	< .001
Ondansetron	+15.1	12.7, 17.5	< .001	+13.3	11.5, 15.2	< .001
Corticosteroids	+13.9	11.0, 16.7	< .001	+10.4	8.2, 12.6	< .001
Dopamine antagonists	+13.2	11.1, 15.4	< .001	+8.4	6.8, 10.1	< .001
NSAIDs	+4.7	2.7, 6.7	< .001	+4.2	2.7, 5.8	< .001
Diphenhydramine	+1.3	−0.9, 3.5	.252	+2.8	1.1, 4.5	.001

Panel B: Other covariates						

Variable	OLS Coefficient (min)	95% CI	*P* value			

Lumbar puncture	+153.5	146.2, 160.8	< .001			
MRI performed	+125.9	119.8, 131.9	< .001			
Admitted/observed	+71.1	68.3, 73.8	< .001			
CT Performed	+44.2	42.4, 46.0	< .001			
Neurology consultation	+41.2	37.3, 45.1	< .001			
Female sex	+3.5	1.7, 5.3	< .001			
Age (per year)	+0.6	0.6, 0.7	< .001			
ESI 1 (vs ESI 3)	−151.1	−158.2, −144.1	< .001			
ESI 2 (vs ESI 3)	−28.8	−31.5, −26.0	< .001			
ESI 4 (vs ESI 3)	−15.2	−17.7, −12.7	< .001			
ESI 5 (vs ESI 3)	−25.2	−35.3, −15.2	< .001			

*CT*, computed tomography; *ED*, emergency department; *ESI*, Emergency Severity Index; *min*, minutes; *MRI*, magnetic resonance imaging; *OLS*, ordinary least squares; *QR*, quantile regression.

**Table 6:** Median length of stay stratified by imaging, procedures, and neurology consultation, in a study of emergency department evaluation and management of headache from 2018–2024.

Procedure/imaging	Median LOS (min)	Number of patients
No Imaging, procedure, or consult	145	43,081
CT imaging only	228	35,161
MRI imaging only	348	454
CT and MRI imaging	414	1,074
Nerve block	338	119
Lumbar Puncture (LP)	444	1,035
Neurology consultation	293	4,283
MRI and neurology consultation	426	594

*CT*, computed tomography; *LOS*, length of stay; min, minutes; *MRI*, magnetic resonance imaging.
