# Supplementary material for: Acute Headache Management and Emergency Department Throughput: A Multicenter Retrospective Analysis
Source: West J Emerg Med. 2026 Sep 24;27(5):1436–46. doi: 10.5811/westjem.61415 (PMC13660621; doi:10.5811/westjem.61415)
Supplement: Supplementary file 1 [file wjem-27-1436-SupplementalTables.pdf]

SUPPLEMENTAL TABLES

Acute Headache Management and Emergency Department Throughput: A Multicenter Retrospective Analysis

Vanood A, Evans BJ IV, Brown SJ, Hodgson NR, Maciulewicz T, Green AL, Martini WA. West J Emerg Med. 2026;27(5). DOI 10.5811/westjem.61415

Supplemental Table S1. Unadjusted associations between medication administration and emergency department length of stay, stratified by headache classification.

Panel A: Primary headache (n = 15,413; median LOS 153 minutes [IQR, 105–224])

Medication	n given	Median LOS given (IQR)	n not given	Median LOS not given (IQR)	Difference (min)	P value
Antiseizure medications	29	283 (210–368)	15,384	153 (105–224)	+130	< .001
IV magnesium	522	246 (196–327)	14,891	150 (104–219)	+96	< .001
Opioids	985	218 (155–310)	14,428	150 (103–218)	+68	< .001
Benzodiazepines	1,004	215 (153–314)	14,409	150 (103–218)	+65	< .001
Acetaminophen	2,066	209 (145–302)	13,347	147 (101–213)	+62	< .001
Antihypertensives	147	214 (158–296)	15,266	153 (105–223)	+61	< .001
IV fluids	10,018	171 (124–242)	5,395	116 (76–181)	+55	< .001
Corticosteroids	2,449	175 (125–255)	12,964	149 (102–219)	+26	< .001
Dopamine antagonists	7,722	167 (115–244)	7,691	142 (97–204)	+25	< .001
Ondansetron	2,417	173 (117–253)	12,996	150 (103–219)	+23	< .001
Diphenhydramine	8,098	157 (110–226)	7,315	149 (100–222)	+8	< .001
Triptans	633	160 (101–249)	14,780	153 (105–223)	+7	.09
NSAIDs	9,895	155 (109–222)	5,518	150 (97–229)	+5	< .001

Panel B: Secondary headache (n = 11,147; median LOS 204 minutes [IQR, 126–317])

Medication	n given	Median LOS given (IQR)	n not given	Median LOS not given (IQR)	Difference (min)	P value
IV magnesium	93	360 (247–462)	11,054	202 (126–315)	+158	< .001
Antiseizure medications	355	325 (213–498)	10,792	200 (124–312)	+125	< .001
Acetaminophen	2,951	271 (178–400)	8,196	183 (115–284)	+88	< .001
Corticosteroids	741	273 (180–407)	10,406	199 (124–311)	+74	< .001
IV fluids	3,012	256 (176–377)	8,135	183 (111–291)	+73	< .001
Dopamine antagonists	1,095	261 (170–398)	10,052	198 (122–309)	+63	< .001
Opioids	2,234	252 (162–376)	8,913	193 (120–301)	+60	< .001
Benzodiazepines	821	255 (159–402)	10,326	200 (124–311)	+55	< .001
Triptans	19	257 (204–402)	11,128	203 (126–317)	+54	.04
Ondansetron	2,319	237 (156–359)	8,828	196 (120–306)	+41	< .001
Diphenhydramine	852	238 (162–360)	10,295	201 (123–313)	+37	< .001
Antihypertensives	1,735	230 (138–353)	9,412	200 (124–311)	+30	< .001
NSAIDs	1,573	207 (143–331)	9,574	202 (123–315)	+5	< .001

Panel C: Unspecified headache (n = 52,967; median LOS 189 minutes [IQR, 123–282])

Medication	n given	Median LOS given (IQR)	n not given	Median LOS not given (IQR)	Difference (min)	P value
Antiseizure medications	248	311 (216–531)	52,719	189 (123–282)	+122	< .001
IV magnesium	843	297 (226–410)	52,124	188 (122–280)	+109	< .001
Opioids	5,948	277 (189–394)	47,019	181 (118–267)	+96	< .001
Antihypertensives	2,223	265 (177–385)	50,744	187 (122–278)	+78	< .001
Benzodiazepines	2,982	264 (183–386)	49,985	186 (121–276)	+78	< .001
Acetaminophen	12,404	237 (158–353)	40,563	177 (116–263)	+60	< .001
IV fluids	23,414	219 (155–312)	29,553	163 (99–256)	+56	< .001
Ondansetron	7,374	231 (155–346)	45,593	183 (119–272)	+48	< .001
Corticosteroids	4,408	220 (149–331)	48,559	187 (121–279)	+34	< .001
Dopamine antagonists	13,914	210 (144–307)	39,053	182 (116–273)	+28	< .001
Triptans	484	204 (131–311)	52,483	189 (123–282)	+14	.006
Diphenhydramine	13,365	197 (137–284)	39,602	187 (118–282)	+10	< .001
NSAIDs	19,533	192 (132–281)	33,434	187 (117–284)	+5	< .001

Results of the Mann-Whitney U test comparing emergency department (ED) length of stay (LOS) by medication administration status within each headache classification. Median LOS with interquartile range (IQR) is shown for encounters where the medication was given versus not given; difference is the median LOS when given minus the median LOS when not given, so a positive difference indicates longer LOS when the medication was administered. Encounters were classified from discharge diagnosis codes: primary headache (migraine, tension-type, cluster, benign headache, and related diagnoses), secondary headache (intracranial hemorrhage, subdural hematoma, subarachnoid hemorrhage, stroke, meningitis, encephalitis, sinusitis, concussion, head injury, COVID-19, and other secondary etiologies), and unspecified headache (headache unspecified, headache not otherwise specified, and diagnoses without a clear primary or secondary classification). Analytic sample: N = 79,527 encounters with LOS > 0 and at most 1,440 minutes, in a study of ED management of headache from 2018–2024. Median LOS differed across the three groups (Kruskal-Wallis H = 1476.85; $P < .001$; all pairwise comparisons $P < .001$ after Bonferroni correction).

ED, emergency department; IQR, interquartile range; IV, intravenous; LOS, length of stay; min, minutes; NSAID, nonsteroidal anti-inflammatory drug.

Supplemental Table S2. Multivariable-adjusted associations between medication administration and emergency department length of stay in the primary headache subgroup (n = 14,634).

Variable	OLS coefficient (min)	95% CI	P value
Antihypertensives	+41.4	27.8, 55.0	< .001
Benzodiazepines	+36.1	30.5, 41.6	< .001
Opioids	+33.6	28.0, 39.2	< .001
Triptans	+30.9	24.3, 37.6	< .001
Acetaminophen	+30.3	26.3, 34.3	< .001
IV magnesium	+30.2	22.3, 38.0	< .001
IV fluids	+25.3	21.9, 28.7	< .001
Ondansetron	+25.2	21.4, 29.0	< .001
Dopamine antagonists	+19.2	16.2, 22.1	< .001
Corticosteroids	+11.2	7.4, 14.9	< .001
Antiseizure medications	+10.4	−19.8, 40.6	.50
Diphenhydramine	+7.6	4.6, 10.6	< .001
NSAIDs	−0.5	−3.7, 2.6	.74

Ordinary least squares (OLS) regression of ED length of stay (minutes) restricted to encounters classified as primary headache with complete covariate data (n = 14,634; R^2 = .47). The model includes all 13 medication classes together with age, sex, Emergency Severity Index level (categorical), disposition, computed tomography, magnetic resonance imaging, neurology consultation, lumbar puncture, site, and year, mirroring the full-cohort model in Table 5. Coefficients are the adjusted difference in LOS (minutes) associated with administration of each medication. Antiseizure medications were not significant in this subgroup, likely because of the very small number of exposed encounters (n = 29).

CI, confidence interval; ED, emergency department; IV, intravenous; LOS, length of stay; min, minutes; NSAID, nonsteroidal anti-inflammatory drug; OLS, ordinary least squares.
